# Impact of CYP2C19 metaboliser status on SSRI response: a retrospective study of 9500 participants of the Australian Genetics of Depression Study

**DOI:** 10.1038/s41397-022-00267-7

**Published:** 2022-01-29

**Authors:** Adrian I. Campos, Enda M. Byrne, Brittany L. Mitchell, Naomi R. Wray, Penelope A. Lind, Julio Licinio, Sarah E. Medland, Nicholas G. Martin, Ian B. Hickie, Miguel E. Rentería

**Affiliations:** 1 QIMR Berghofer Medical Research Institute, Brisbane, QLD Australia; 2 Faculty of Medicine, The University of Queensland, Brisbane, QLD Australia; 3 Institute for Molecular Bioscience, The University of Queensland, Brisbane, QLD Australia; 4 School of Biomedical Sciences, Queensland University of Technology, Brisbane, QLD Australia; 5 Queensland Brain Institute, The University of Queensland, Brisbane, QLD Australia; 6 Department of Psychiatry, State University of New York Upstate Medical University, Syracuse, NY USA; 7 Brain and Mind Centre, University of Sydney, Camperdown, NSW Australia

**Keywords:** Genetics research, Predictive markers

## Abstract

**Background:**

Variation within the *CYP2C19* gene has been linked to differential metabolism of selective serotonin reuptake inhibitors (SSRIs). Pharmacogenetic recommendations based on the effect of *CYP2C19* variants have been made available and are used increasingly by clinical practitioners. Nonetheless, the underlying assumption linking differential metabolism to efficacy or adverse side effects remains understudied. Here, we aim to fill this gap by studying *CYP2C19* polymorphisms and inferred metabolism and patient-reported antidepressant response in a sample of 9531 Australian adults who have taken SSRIs.

**Methods:**

Metaboliser status was inferred for participants based on *CYP2C19* alleles. Primary analysis consisted of assessing differences in treatment efficacy and tolerability between *normal* (reference) and: *ultrarapid*, *rapid*, *intermediate* and *poor* metabolisers.

**Results:**

Across medications, poor metabolisers reported a higher efficacy, whereas rapid metabolisers reported higher tolerability. When stratified by drug, associations between metaboliser status and efficacy did not survive multiple testing correction. Intermediate metabolisers were at greater odds of reporting any side effect for sertraline and higher number of side effects across medications and for sertraline.

**Conclusions:**

The effects between metaboliser status and treatment efficacy, tolerability and side effects were in the expected direction. Our power analysis suggests we would detect moderate to large effects, at least nominally. Reduced power may also be explained by heterogeneity in antidepressant dosages or concomitant medications, which we did not measure. The fact that we identify slower metabolisers to be at higher risk of side effects even without adjusting for clinical titration, and the nominally significant associations consistent with the expected metabolic effects provide new evidence for the link between CYP2C19 metabolism and SSRI response. Nonetheless, longitudinal and interventional designs such as randomized clinical trials that stratify by metaboliser status are necessary to establish the effects of *CYP2C19* metabolism on SSRI treatment efficacy or adverse effects.

## Introduction

Major Depressive Disorder (MDD) is one of the leading causes of disability and an important contributor to the burden of disease worldwide [1, 2]. MDD of at least moderate severity or persistence is commonly treated with antidepressant medication. While recent reports support antidepressant efficacy for treating depression [3], a significant proportion of people do not respond optimally or cease treatment due to early adverse effects [4, 5]. Several classes of antidepressants exist but, even within the same class, treatment response is heterogeneous, and people can experience a range of side-effects which affect tolerability and treatment adherence. For example, although selective serotonin reuptake inhibitors (SSRIs) are the preferred MDD treatment, at least one in three people might not respond to SSRI treatment [4].

Etiological heterogeneity might contribute to individual differences in treatment response [6] in depression. For instance, comorbid chronic pain, melancholic and anxious depression subtypes have been associated with lower antidepressant response as measured by efficacy and remission [7–9]. Genetic susceptibility also plays a role in antidepressant treatment response [10]. Biomarker studies have identified some candidate pathways [11] and genes [12] potentially associated with differential treatment response. For example, the cytochrome P450 2C19 (*CYP2C19)* gene is known to encode a drug-metabolizing enzyme which has been shown to metabolize the SSRIs sertraline, citalopram and escitalopram [13, 14] among other drugs.

Pharmacogenetic recommendations based on *CYP2C19* polymorphisms are commercially available and increasingly used by clinicians. In fact, direct-to-consumer genetic service companies have received FDA clearance to report these recommendations [15, 16]. The current Clinical Pharmacogenetics Implementation Consortium (CPIC) guidelines report poor metabolisers to be at increased risk of adverse side-effects due to higher antidepressant serum concentrations [13, 17, 18]. Ultrarapid metabolisers, on the other hand, are hypothesized to be more likely to fail therapy due to a decreased exposure, and the current recommendation is to switch to an alternative SSRI not metabolized by CYP2C19 [13]. The main assumption behind these recommendations is that differential metabolism will result in differential treatment efficacy or side-effects due to under- or over-exposure to the active drug [18].

There is a clear effect of CYP2C19 variation on SSRI metabolism. For example, a recent analysis on 1200 participants identified higher concentrations of sertraline in poor and intermediate metabolisers and slightly lower concentrations in ultrarapid compared to normal metabolisers [18]. Thus, a link between differential metabolism and differential response is a plausible assumption. However, whether genetic variants that modify CYP2C19 activity are linked to treatment efficacy or side effects is a question that remains understudied. Studies assessing a link between *CYP2C19* genotype and treatment response, tolerance or adverse effects have reached inconsistent conclusions. A previous study [19] identified no evidence for an association with *CYP2C19* polymorphisms, whereas another one [20] concluded that *CYP2C19* variants associate with tolerance and remission using the same sample (STAR*D). A recent publication [21] described evidence of CYP2C19 metaboliser status to be associated with switching from escitalopram to any other antidepressant, which was considered a proxy for therapeutic failure. Furthermore, the GENDEP study performed a comprehensive analysis measuring both serum escitalopram concentration and treatment response. While *CYP2C19* variation was indeed associated with escitalopram and desmethylcitalopram serum concentrations, neither the genotypes nor the measured concentrations were associated with treatment response [22]. Although a study has identified increased adverse side effects and lower treatment tolerability for slower CYP2C19 metabolisers [23], another one identified no association between history of tolerability and CYP2C19 [24]. Notably, most of these studies included samples of relatively small size and thus, were prone to false-positive associations and lacked sufficient statistical power. Although there are examples of recent studies with moderate sample sizes (ranging from ~1200 to ~2000 participants), these have directly focused only on substrate concentrations without assessing treatment response [18], or used proxy measures for therapeutic failure [21]. These compromises are understandable given the difficulty of performing complete pharmacogenomic studies (i.e., measuring response, side effects and drug concentrations longitudinally) in large samples. We thus argue that convergent evidence from studies with distinct designs will help advance the field.

The present study aims to assess the association between CYP2C19 SSRI inferred metaboliser status based on individual *CYP2C19* polymorphisms and patient-reported efficacy of sertraline, citalopram and escitalopram in the Australian Genetics of Depression Study (AGDS). We explore whether those participants harboring diplotypes associated with ultrarapid, rapid, intermediate and poor CYPC2C19 metabolism display differential treatment efficacy, tolerability or side-effects compared to normal metabolisers. Our study provides novel insights and convergent evidence on the associations between CYP2C19 metabolism and SSRI treatment response in a large well-powered data set.

## Methods

### Sample recruitment

The AGDS sample recruitment has been previously discussed in detail [5]. Briefly, two strategies were employed: (i) a mail out by the Australian Department of Human Services (DHS) targeting people who had been prescribed antidepressants in the past 4.5 years and (ii) a national media publicity campaign for people who had been “diagnosed with depression by a doctor, psychiatrist or psychologist”. Only 14.3% of participants were recruited through the DHS. The DHS carried out its own ethics approval and did not share at any time identifying information with the study researchers. Interested participants were directed to a website where they provided informed consent prior to participating through online questionnaires. Upon completion of the core questionnaire, participants were mailed a GeneFix GFX-02 2 mL saliva DNA extraction kit (Isohelix plc) to use at home and post for genotyping. All protocols and questionnaires were approved by the QIMR Human Research Ethics Committee under project number 1128.

### Phenotype ascertainment

This study focuses on patient-reported antidepressant efficacy and adverse side effects (ASE). Participants were first asked to confirm whether they had ever been prescribed any of the ten most commonly prescribed antidepressants in Australia (sertraline, escitalopram, venlafaxine, fluoxetine, citalopram, desvenlafaxine, duloxetine, mirtazapine, amitriptyline and paroxetine). For each antidepressant taken, participants were asked to rate their perceived effectiveness using the item: “How well does/did [name of the antidepressant] work for you?”; the possible responses were: ‘not at all well’ (1), ‘moderately well’ (2) and ‘very well’ (3). Similarly, for each antidepressant taken, participants were asked whether they had experienced side effects. Tolerability was measured using the item: “*Did you have to stop taking any antidepressant because of side effects?*”. Binary responses (yes/no) were collected for each antidepressant. In this study we focus on the SSRIs: sertraline, escitalopram and citalopram as they are reported to be extensively metabolized by CYP2C19 [13]. Participants who failed to report a diagnosis of major depressive disorder (~5%) were excluded from this study. CYP2C19 metaboliser status was inferred based on the CPIC guidelines [25, 26]. Briefly, participants were categorized into poor, intermediate, normal, rapid and ultrarapid CYP2C19 metabolisers based on their combination of *CYP2C19* alleles (see Supplementary Table S1).

#### Genotyping and QC

Genotyping was performed using the Illumina Global Screening Array (GSA V.2.0.) across three genotyping centers. A common set of high-quality markers between the different genotyping centers was identified prior to joint imputation. Pre-imputation marker exclusion criteria consisted of unknown or ambiguous map position and strand alignment in a BLAST search, missingness > 5%, Hardy–Weinberg equilibrium test *p* < 10^−6^), minor allele frequency < 1%, and GenTrain score < 0.6. The Michigan imputation server was used to impute the genotypes using the HRCr1.1 reference panel. Individuals were excluded based on a high missingness (missing rate > 3%), inconsistent (and unresolvable) sex, or if deemed ancestry outliers from the European population (6 standard deviations from the centroid of the first two genetic principal components). Imputed dosages for three *CYP2C19* polymorphisms (rs12248560, rs4244285 and rs4986893) were transformed to hard calls using PLINK1.9. The imputation quality for the *CYP2C19* polymorphisms was high (INFO > 0.90) and the allele frequencies for the European Ancestry subpopulation were consistent with previous reports [26]. These alleles represent the minimum panel of variant alleles (tier 1) recommended by the Association for Molecular Pathology (AMP) for *CYP2C19* pharmacogenomic allele selection [27]. Notably, these are also the *CYP2C19* genetic variants that 23andMe uses to base their recently cleared pharmacogenetic reports [16].

#### Statistical analyses

All analyses were performed on a subset of unrelated participants (genetic relatedness < 0.05) of European Ancestry. Logistic regressions were used to assess the association between *CYP2C19* inferred metaboliser status and binary phenotypes such as side-effects. Cumulative linked models (ordered logistic regressions) were used to assess the association between ordinal SSRI efficacy variables and CYP2C19 inferred metaboliser status. All associations were adjusted for age (at recruitment) and sex. For the associations pooling data across sertraline, escitalopram and citalopram (i.e., those labeled *All* in the results) mixed effects models were used to account for repeated measures (i.e., participants that took more than one SSRI) by including a random intercept conditional on each participant ID while adjusting for antidepressant, age and sex. All analyses were performed in R using the *base, lme4, tidyr* and *ordinal* libraries [28–30]. The statistical significance threshold was defined using a Bonferroni correction (0.05/n) for multiple testing adjusting for the number of comparisons performed within each of the studied outcomes. That is, for the main analysis assessing efficacy, the corrected significance threshold was *p* < 0.0125 (4 comparisons of each group against the normal metaboliser status). When assessing the association between metaboliser status and side effects the corrected significance threshold was *p* < 0.05/300 (25 side effects; 3 antidepressants; 4 metaboliser status).

#### Power calculation

A *post-hoc* power calculation was performed to estimate the effect sizes the metaboliser categories should have for this study to have an 80% power to detect them at a nominally significant threshold of *p* < 0.05. Briefly, two linear equations describing the logarithm of the odds for the two thresholds were generated. The first one describes the log of the sum of probabilities of being a category 2 (moderate) or 3 (very well) over the probability of being 1 (poor) for antidepressant efficacy. The second one described the log of the sum of probabilities of being a good responder (i.e., score of 3) over the log of the sum of probabilities of being poor or moderate responder. These linear equations include a specific intercept (following the proportional odds assumption), and a set of common parameters reflecting the effects of age, sex and the different metaboliser statuses. We varied the effects of the different metaboliser statuses over a range of effect sizes (ORs ~1.05, 1.1, 1.16, 1.22, 1.5, 1.65 and 1.82). We then used the inverse logit function to derive the probabilities of each group for each observation and used these probabilities to generate a sample of simulated antidepressant efficacy scores. Finally, we performed the ordered logistic regression as described above to assess whether we were able to detect the effect size with nominal significance (*p* < 0.05). This procedure was repeated 1000 times and the power for each effect size was estimated as the number of times we rejected the null hypothesis of no effect over the number of trials.

## Results

### Metaboliser and CYP2C19 allele prevalence

Our sample comprised 9,531 unrelated individuals of European ancestry with genotype and phenotype data and a diagnosis of depression. Most participants (*N* = 3,869, 40.6%) had *CYP2C19* alleles that would be interpreted as *CYP2C19* normal metabolisers. As expected, poor (*N* = 199, 2.1%) and ultra-rapid metabolisers (*N* = 448, 4.7%) were the least common groups; followed by intermediate (*N* = 2460, 25.8%) and rapid (*N* = 2555, 26.8%) metabolisers (Table 1 and Supplementary Fig. S1). Minor allele frequencies for the studied variants were 21.4% for CYP2C19*17 (rs12248560), 14% for CYP2C19*2 (rs4244285) and 0.02% for CYP2C19*3 (rs4986893). After removing participants with missing data on treatment response, 9168 participants remained. Most participants reported taking sertraline followed by citalopram and escitalopram respectively (Table 1). Around 25% of participants reported taking two antidepressants and only 6% reported taking all three (Supplementary Fig. S2).

**Table 1:** Sample demographics and distribution of metaboliser status and antidepressant intake.

Metaboliser	*N*	Age (s.d)	Sex (F)	N Sertraline^a^	N Citalopram^a^	N Escitalopram^a^
Poor	199	42.6 (15.1)	153	123	51	76
Intermediate	2460	43 (14.5)	1925	1479	673	1115
Normal	3869	42.8 (15.1)	2918	2320	965	1762
Rapid	2555	43.1 (14.2)	1985	1493	703	1201
Ultrarapid	448	42.6 (14.0)	350	265	111	188

^a^Sample sizes across antidepressants do not add to the total sample size as some participants reported taking more than one antidepressant. Repeated measures were dealt with statistically (see methods).

### Efficacy

**Fig. 1:**
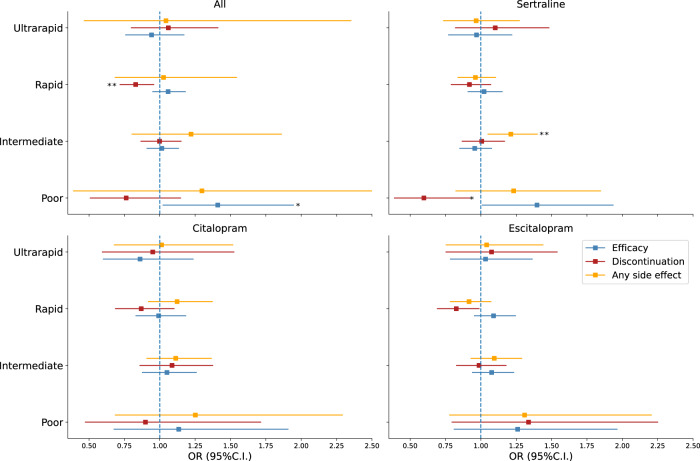
Inferred metaboliser status association with treatment response. Forest plots depict odds ratios and 95% confidence intervals for the association between metaboliser category (compared to normal) and treatment efficacy (blue markers) treatment discontinuation due to side effects as a measure inverse to tolerability (red markers) or experiencing any side-effects (orange markers). *All* represents the results of a mixed effects model testing for association between the outcome of interest and the pooled response variables across sertraline, citalopram and escitalopram. *p < 0.05 **p significant after correction for multiple testing.

We first assessed whether CYP2C19 metaboliser status was associated with differential efficacy across any of the SSRIs under study. Table 2 shows the distribution of self-reported antidepressant efficacy per antidepressant and metaboliser status. Overall, poor metabolisers showed a nominally significant higher antidepressant efficacy (OR = 1.41 [1.02–1.95] *p* = 0.037). A similar result was observed for sertraline (OR = 1.40 [1.01–1.94] *p* = 0.045), but not for escitalopram or citalopram. Associations between metaboliser status and efficacy did not reach significance after adjusting for multiple testing (*p* < 0.0125; Supplementary Table S2). Despite a lack of significant differences, a clear trend between predicted slower CYP2C19 metabolism and higher citalopram efficacy was observed (Fig. 1c). A similar pattern, although not as clear, was also observed for the other medications.

**Table 2:** Antidepressant response by CYP2C19 metaboliser status.

	Low	Moderate	Well
Metaboliser status	Sertraline (*N* = 5680)		
Poor	32 (26%)	52 (42%)	39 (32%)
Intermediate	543 (37%)	551 (37%)	385 (26%)
Normal	851 (37%)	825 (36%)	644 (28%)
Rapid	543 (36%)	520 (35%)	430 (29%)
Ultrarapid	93 (35%)	107 (40%)	65 (24%)
	Citalopram (*N* = 2503)		
Poor	18 (35%)	19 (37%)	14 (27%)
Intermediate	251 (37%)	232 (34%)	190 (28%)
Normal	349 (36%)	376 (39%)	240 (25%)
Rapid	258 (37%)	266 (38%)	179 (25%)
Ultrarapid	45 (41%)	41 (37%)	25 (22%)
	Escitalopram (*N* = 4342)		
Poor	26 (34%)	18 (24%)	32 (42%)
Intermediate	339 (30%)	425 (38%)	351 (31%)
Normal	564 (32%)	672 (38%)	526 (30%)
Rapid	369 (31%)	444 (37%)	388 (32%)
Ultrarapid	61 (32%)	66 (35%)	61 (32%)

Percentages are presented conditional on antidepressant and metaboliser status (e.g., 26% of poor metabolisers taking sertraline reported a low efficacy).

### Tolerability

We then tested whether CYP2C19 metaboliser status were associated with treatment tolerability. Within participants with genetic and tolerability data, 1885 (45%) 1188 (37%) and 764 (44%) stopped taking sertraline, escitalopram and citalopram due to side effects respectively (Table 3). A trend whereby rapid metabolisers were less likely to stop medication due to side-effects was observed (Fig. 1). Across medications, rapid metabolisers had a higher tolerability (OR for stopping medication use due to side-effects = 0.83 [0.72–0.96] *p* < 0.0125) compared to normal metabolisers. Furthermore, two nominally significant associations between tolerability and metaboliser status were identified. Rapid CYP2C19 metabolisers showed higher tolerability for escitalopram (OR = 0.83 [0.69–0.99] *p* < 0.05) and poor metabolisers showed a higher tolerability for sertraline compared to normal metabolisers (OR = 0.60 [0.39–0.92] *p* < 0.05; Fig. 1 and Supplementary Table S2).

**Table 3:** Discontinuation and side effects by metaboliser status.

	Discontinutation No. (%)		Any side effect No. (%)	
	No	Yes	Yes	No
Sertraline				
Poor	63 (66)	32 (34)	98 (74)	34 (26)
Intermediate	610 (54)	519 (46)	1121 (73)	405 (27)
Normal	917 (54)	775 (46)	1683 (70)	716 (30)
Rapid	598 (56)	466 (44)	1069 (69)	480 (31)
Ultrarapid	100 (52)	93 (48)	190 (70)	83 (30)
Citalopram				
Poor	23 (57)	17 (43)	39 (71)	16 (30)
Intermediate	256 (53)	224 (47)	478 (67)	232 (33)
Normal	360 (55)	290 (45)	656 (65)	356 (35)
Rapid	285 (59)	199 (41)	501 (67)	246 (33)
Ultrarapid	44 (56)	34 (44)	78 (65)	41 (34)
Escitalopram				
Poor	33 (55)	27 (45)	60 (75)	20 (25)
Intermediate	522 (62)	322 (38)	846 (73)	319 (27)
Normal	791 (62)	493 (38)	1300 (71)	534 (29)
Rapid	562 (66)	291 (34)	857 (69)	385 (31)
Ultrarapid	82 (60)	55 (40)	142 (71)	58 (29)

### Side effects

Finally, we tested whether metaboliser status was associated with medication adverse side effects. To reduce multiple testing burden, we focused on reporting any side effect (Table 3). Compared to normal, intermediate metabolisers showed greater odds of reporting any side effect for sertraline (OR = 1.23 [1.08–1.41]; *p* = 0.009). Associations with escitalopram or citalopram did not reach statistical significance (Fig. 1). As secondary analyses, we tested for association between metaboliser status and (i) number of side effects or (ii) reporting 23 specific side effects. A similar pattern of associations to *any side effects* was observed for number of side effects. Intermediate metabolisers were at increased odds of reporting more side effects across drugs (*p* = 0.005) and for sertraline (*p* = 0.002 Supplementary Table S3). When testing for specific side effects, no results survived our defined multiple testing corrected threshold (*p* < 0.00016). Nonetheless, fifteen nominally significant associations were identified (Supplementary Table S4). The nominally significant associations (*p* < 0.05) were enriched for slower (intermediate or poor) metabolisers having increased risk for side effects. The three most significant associations were between intermediate metabolisers (compared to normal metabolisers) and sertraline side effects (namely, weight loss OR = 2 [1.37–3.05], fatigue OR = 1.36 [1.35–1.63] and drowsiness OR = 1.3 [1.07–1.53]; Supplementary Fig. S3).

### Power analysis

We performed a power analysis based on simulations (see methods). Our results indicated we had the most power to detect nominally significant associations (*p* < 0.05) between antidepressant efficacy and intermediate or rapid metaboliser status. Power to detect these associations with poor and ultra-rapid metaboliser status was lower. We estimate our study to have >80% power to detect odds ratios across medications higher than 0.45 (i.e., 1.45 or 0.69), 0.3, 0.14 and 0.14 for the poor, ultra-rapid intermediate and rapid metaboliser groups respectively (Supplementary Fig. S4).

## Discussion

Here, we leveraged data from the AGDS to assess whether differential metabolism of sertraline, citalopram or escitalopram was linked to treatment efficacy, tolerability or side effects. This is, to the best of our knowledge, the largest study on this subject to date. CYP2C19 polymorphism allele frequencies in the AGDS were highly concordant with recent estimates obtained from a European subset of 23andMe customers [26]. We showed that CYP2C19 intermediate metabolisers had higher odds of reporting more side effects both across medications and specifically for sertraline. Our results are consistent with the hypothesis that slower (intermediate) metabolisers are at increased risk of adverse side effects. Notably, the hypothesized pattern of lower tolerability and higher side effects for poor metabolisers was not observed, this is likely explained by the small size of the poor metaboliser group. Our study did not identify statistically significant associations between inferred metaboliser status and treatment efficacy or tolerability. Nonetheless, a pattern whereby faster metabolisers reported lower efficacy and slower metabolisers reported a higher efficacy was observed.

Overall, our results provide some evidence of a relationship between treatment response and CYP2C19 metaboliser status, but several expected associations did not reach statistical significance. While the multiple testing burden might explain this, other plausible hypotheses could underlie our results. First, CYP2C19 is not the only drug-metabolizing enzyme for these compounds, and differential CYP2C19 metabolism would not affect all drugs equally. Furthermore, differential metabolism of sertraline, citalopram or escitalopram could be independent of a treatment efficacy, or have a moderate effect size, which we were not powered to detect. This would be consistent with known effect sizes of common genetic variants on complex traits. We also cannot rule out possible downstream compensatory mechanisms potentially normalizing the concentration of the active compounds in the brain. Although this would be inconsistent with titration and dosage effects, most studies have suggested a lack of any clear dose-dependent effects for sertraline [31]. Finally, it is likely that efficacy is associated with etiological factors contributing to MDD heterogeneity such as differential causative paths, concurrent physical illness, comorbid alcohol or other substance misuse. Finally, sertraline, citalopram, and escitalopram have wide therapeutic windows, with a large range between effective and toxic drug concentrations; therefore, small to moderate variations in drug availability caused by *CYP2C19* polymorphisms might not have been enough to result in changes in treatment response.

Some limitations of the present study need to be considered. First, this is a retrospective study. Patient-reported measures of antidepressant efficacy and side effects are subject to recall bias and heterogeneous definitions. The AGDS did not collect specific information on the antidepressant dosages, which would be ideal to consider concerning metaboliser status. Clinicians will usually adjust dosages based on patient characteristics and, *a posteriori*, response and side effects. Furthermore, several substances and medications are known to inhibit, induce or be metabolized by CYP2C19. We did not gather detailed information on, and could not account for, concomitant medication or substance use. These two factors (regime changes and phenoconversion) would increase heterogeneity and reduce our ability to identify meaningful associations. The fact that we identified a significant association between slower metabolism and side effects could be argued as evidence that titration is affecting our measures of side effects to a lesser degree compared to measures of efficacy. This is expected as efficacy is measured by a broader construct that could encompass distinct factors such as symptom alleviation, side effect profile and long-term outcomes. Moreover, we focused on the three most common *CYP2C19* alleles; rarer alleles exist but typing or imputing them remains challenging. However, these are the minimum panel of variant alleles (tier 1) recommended by the AMP [27] and they are the variants that 23andMe uses for their pharmacogenetic consumer reports [16].

Furthermore, to avoid potential confounding from population stratification and relatedness, we have focused on a subset from the AGDS consisting of unrelated participants of European ancestry. Therefore, caution must be taken when generalizing our observations to other populations. As a retrospective study, we were incapable of assessing serum drug concentrations, which would have given a more precise correlation among clinical picture, drug bioavailability and *CYP2C19* polymorphisms. Most of these limitations are linked to our study design, which aims at maximizing sample size to identify subtle effects associated with depression outcomes. Such an increase in sample size usually comes with limited phenotyping ability. Nonetheless, the AGDS represents a unique dataset in that detailed patient-reported outcomes for diagnosis ascertainment and treatment response including efficacy and adverse side effects have been collected.

Overall, we found evidence for an association between slower CYP2C19 metabolism and adverse side effects. This result is consistent with current guidelines and hypothesized effects. We also observed the expected direction of effects between metaboliser status and treatment response. However, most of these associations did not reach statistical significance after accounting for multiple testing correction. Given the size of our sample, we would expect to have enough power to identify moderate effects of metaboliser status. Nonetheless, reduced power could be expected from distinct drug exposures due to dosage adjustments, which could not account for in this study. Therefore, pharmacogenomic studies should focus on increasing sample sizes and implementing interventional or longitudinal studies sufficiently powered to assess whether metaboliser status is not only statistically but also clinically relevant to treatment with SSRIs.

## Supplementary information

Supplementary Figures

Supplementary Table S1

Supplementary Table S2

Supplementary Table S3

Supplementary Table S4

## Data Availability

Code used for this study are publically available through a Zenodo repository online 10.5281/zenodo.5834893.

## References

[1] Lopez AD, J. CC (1998). The global burden of disease, 1990–2020. Nat Med.

[2] Vigo D, Thornicroft G, Atun R (2016). Estimating the true global burden of mental illness. Lancet Psychiatry.

[3] Cipriani A, Furukawa TA, Salanti G, Chaimani A, Atkinson LZ, Ogawa Y (2018). Comparative efficacy and acceptability of 21 antidepressant drugs for the acute treatment of adults with major depressive disorder: a systematic review and network meta-analysis. Lancet.

[4] Fredman SJ, Fava M, Kienke AS, White CN, Nierenberg AA, Rosenbaum JF (2000). Partial response, nonresponse, and relapse with selective serotonin reuptake inhibitors in major depression: a survey of current “next-step” practices. J Clin Psychiatry.

[5] Byrne EM, Kirk KM, Medland SE, McGrath JJ, Colodro-Conde L, Parker R (2020). Cohort profile: the Australian genetics of depression study. BMJ Open.

[6] Kessler RC, van Loo HM, Wardenaar KJ, Bossarte RM, Brenner LA, Ebert DD (2017). Using patient self-reports to study heterogeneity of treatment effects in major depressive disorder. Epidemiol Psychiatr Sci.

[7] Fava M, Uebelacker LA, Alpert JE, Nierenberg AA, Pava JA, Rosenbaum JF (1997). Major depressive subtypes and treatment response. Biol Psychiatry.

[8] Fava M, Rush AJ, Alpert JE, Balasubramani GK, Wisniewski SR, Carmin CN (2008). Difference in treatment outcome in outpatients with anxious versus nonanxious depression: a STAR*D report. Am J Psychiatry.

[9] Roughan WH, Campos AI, García-Marín LM, Cuéllar-Partida G, Lupton MK, Hickie IB (2021). Comorbid Chronic Pain and Depression: Shared Risk Factors and Differential Antidepressant Effectiveness. Front Psychiatry.

[10] Campos AI, Mulcahy A, Thorp JG, Wray NR, Byrne EM, Lind PA (2021). Understanding genetic risk factors for common side effects of antidepressant medications. Commun Med.

[11] Park DI, Dournes C, Sillaber I, Uhr M, Asara JM, Gassen NC (2016). Purine and pyrimidine metabolism: convergent evidence on chronic antidepressant treatment response in mice and humans. Sci Rep.

[12] Licinio J, O’Kirwan F, Irizarry K, Merriman B, Thakur S, Jepson R (2004). Association of a corticotropin-releasing hormone receptor 1 haplotype and antidepressant treatment response in Mexican-Americans. Mol Psychiatry.

[13] Hicks JK, Bishop JR, Sangkuhl K, Müller DJ, Ji Y, Leckband SG (2015). Clinical Pharmacogenetics Implementation Consortium (CPIC) guideline for CYP2D6 and CYP2C19 genotypes and dosing of selective serotonin reuptake inhibitors. Clin Pharm Ther.

[14] Bank PCD, Caudle KE, Swen JJ, Gammal RS, Whirl‐Carrillo M, Klein TE (2018). Comparison of the Guidelines of the Clinical Pharmacogenetics Implementation Consortium and the Dutch Pharmacogenetics Working Group. Clin Pharm Ther.

[15] Genome Web. 23andMe Garners FDA Clearance for CYP2C19 PGx Test Report. 2020. Available online at: https://www.genomeweb.com/regulatory-news-fda-approvals/23andme-garners-fda-clearance-cyp2c19-pgx-test-report#.YfHN7PVBzWY.

[16] FDA. 510(k) substantial equivalence determination decision summary (K193492). Department of Health and Human Services. FDA. 2020. Available online at: https://www.accessdata.fda.gov/cdrh_docs/pdf19/K193492.pdf

[17] Furukawa TA, Cipriani A, Cowen PJ, Leucht S, Egger M, Salanti G (2019). Optimal dose of selective serotonin reuptake inhibitors, venlafaxine, and mirtazapine in major depression: a systematic review and dose-response meta-analysis. Lancet Psychiatry.

[18] Bråten LS, Haslemo T, Jukic MM, Ingelman-Sundberg M, Molden E, Kringen MK (2020). Impact of CYP2C19 genotype on sertraline exposure in 1200 Scandinavian patients. Neuropsychopharmacology.

[19] Peters EJ, Slager SL, Kraft JB, Jenkins GD, Reinalda MS, McGrath PJ (2008). Pharmacokinetic genes do not influence response or tolerance to citalopram in the STAR*D sample. PLoS One.

[20] Mrazek DA, Biernacka JM, O’Kane DJ, Black JL, Cunningham JM, Drews MS (2011). CYP2C19 variation and citalopram response. Pharmacogenet Genom.

[21] Jukić MM, Haslemo T, Molden E, Ingelman-Sundberg M (2018). Impact of CYP2C19 genotype on escitalopram exposure and therapeutic failure: a retrospective study based on 2,087 patients. Am J Psychiatry.

[22] Hodgson K, Tansey K, Dernovsek MZ, Hauser J, Henigsberg N, Maier W (2014). Genetic differences in cytochrome P450 enzymes and antidepressant treatment response. J Psychopharmacol.

[23] Aldrich SL, Poweleit EA, Prows CA, Martin LJ, Strawn JR, Ramsey LB (2019). Influence of CYP2C19 metabolizer status on escitalopram/citalopram tolerability and response in youth with anxiety and depressive disorders. Front Pharm.

[24] Maggo S, Kennedy MA, Barczyk ZA, Miller AL, Rucklidge JJ, Mulder RT (2019). Common CYP2D6, CYP2C9, and CYP2C19 gene variants, health anxiety, and neuroticism are not associated with self-reported antidepressant side effects. Front Genet.

[25] Hicks JK, Sangkuhl K, Swen JJ, Ellingrod VL, Müller DJ, Shimoda K (2017). Clinical pharmacogenetics implementation consortium guideline (CPIC) for CYP2D6 and CYP2C19 genotypes and dosing of tricyclic antidepressants: 2016 update. Clin Pharmacol Therapeutics.

[26] Ionova Y, Ashenhurst J, Zhan J, Nhan H, Kosinski C, Tamraz B (2020). CYP2C19 allele frequencies in over 2.2 million direct-to-consumer genetics research participants and the potential implication for prescriptions in a large health system. Clin Transl Sci..

[27] Pratt VM, Del Tredici AL, Hachad H, Ji Y, Kalman LV, Scott SA (2018). Recommendations for clinical CYP2C19 genotyping allele selection: a report of the association for molecular pathology. J Mol Diagnostics.

[28] R Core Team. R: A language and environment for statistical computing. R Foundation for Statistical Computing, Vienna, Austria. 2018. Available online at https://www.R-project.org/.

[29] Bates D, Mächler M, Bolker B, Walker S (2015). Fitting linear mixed-effects models using lme4. J Stat Softw.

[30] Christensen, R. H. B. Ordinal—regression models for ordinal data. 2011 R package available online at: http://www.cran.r-project.org/package=ordinal/.

[31] Kato T, Furukawa TA, Mantani A, Kurata KI, Kubouchi H, Hirota S (2018). Optimising first- and second-line treatment strategies for untreated major depressive disorder - the SUND study: a pragmatic, multi-centre, assessor-blinded randomised controlled trial. BMC Med.

