# Supplementary material for: Impact of CYP2C19 metaboliser status on SSRI response: a retrospective study of 9500 participants of the Australian Genetics of Depression Study
Source: Pharmacogenomics J. 2022 Jan 29;22(2):130–5. doi: 10.1038/s41397-022-00267-7 (PMC8975743; doi:10.1038/s41397-022-00267-7)
Supplement: Supplementary file 1 — Supplementary Figures [file 41397_2022_267_MOESM1_ESM.pdf]

**Supplementary Material**

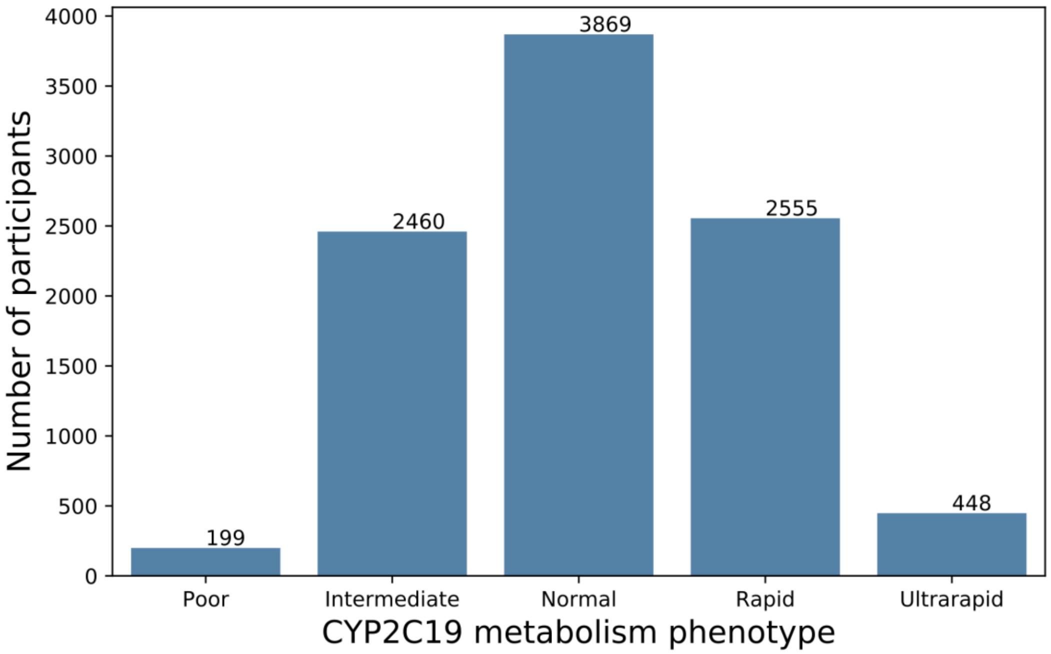


**Supplementary Figure S1** Histogram of participant CYP2C19 metabolizer status

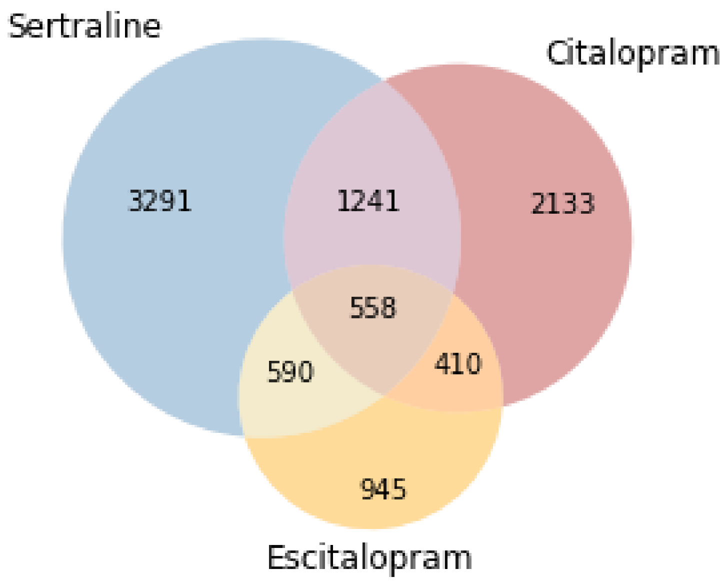


**Supplementary Figure S2**. Venn diagram showing the participant overlap across the SSRIs under study. Numbers represents participants with non-missing observations on efficacy and treatment response

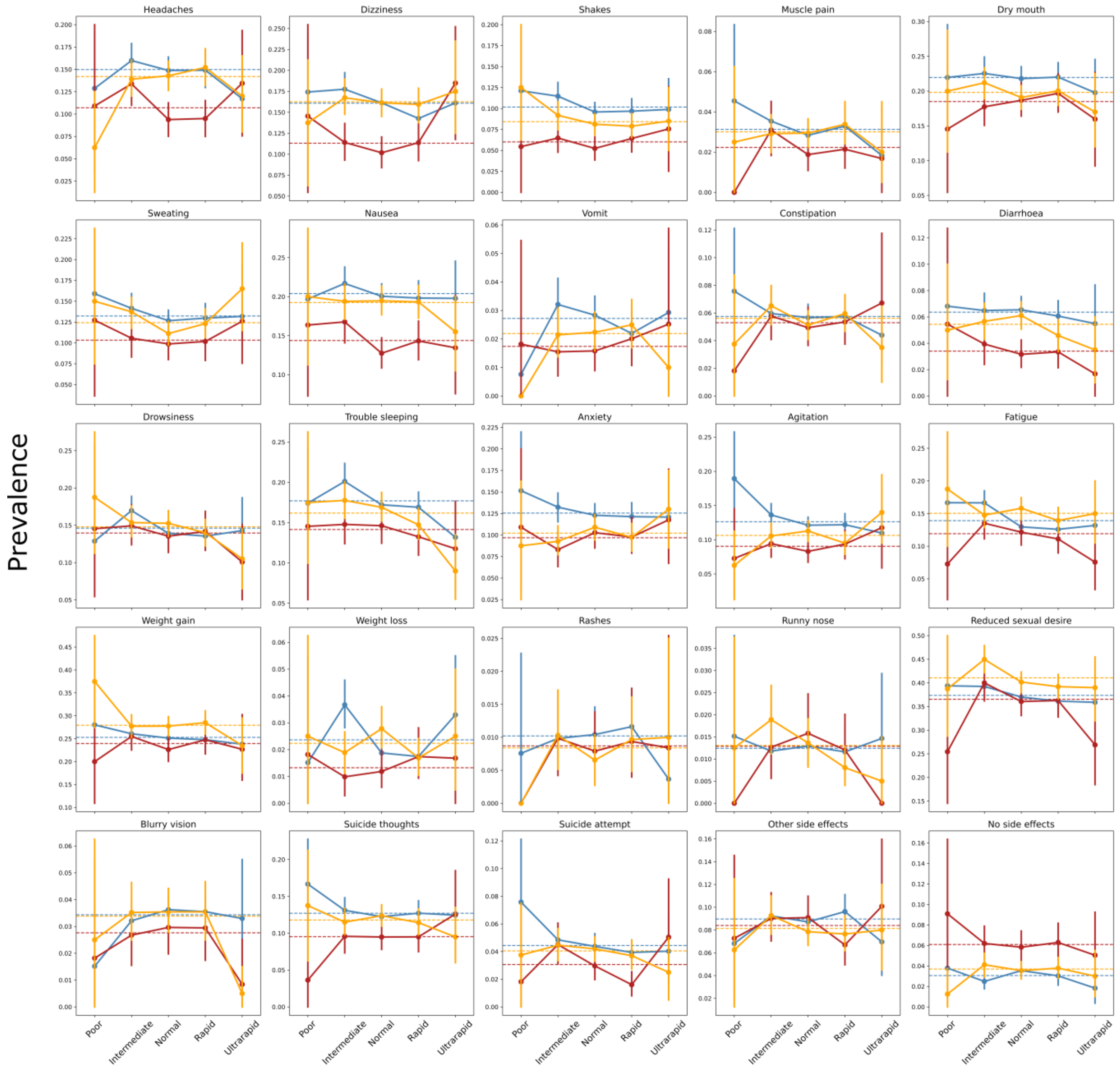


**Supplementary Figure S3**. Prevalence of studied side effects from sertraline (blue), escitalopram (orange) and citalopram (red) across metaboliser groups.

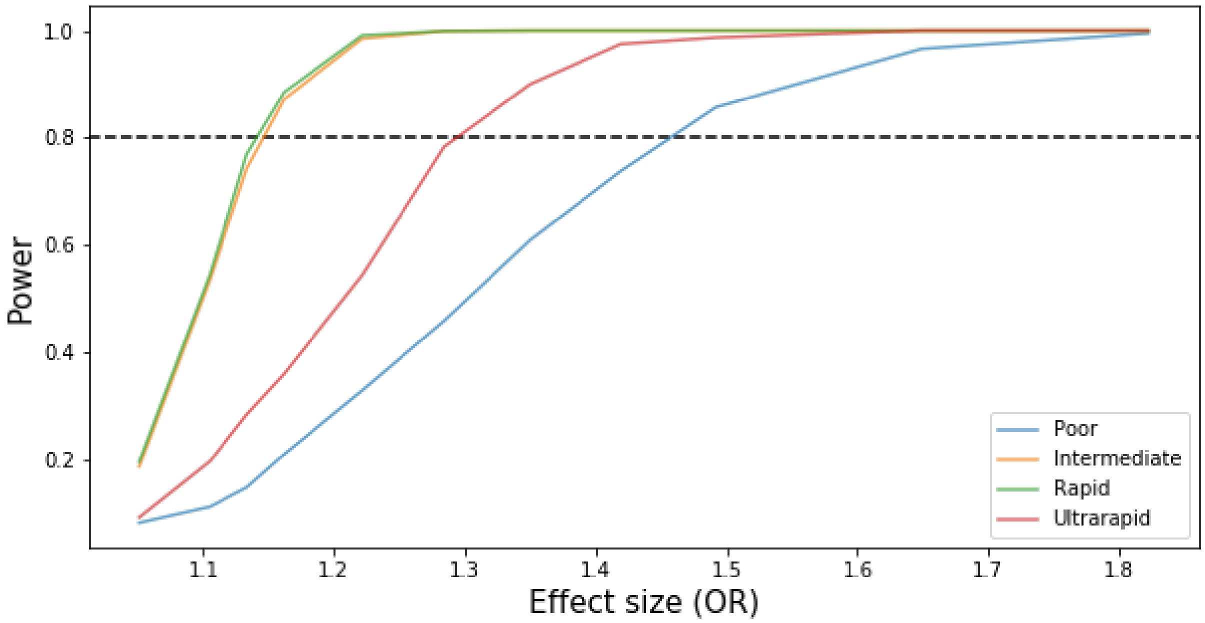


**Supplementary Figure S4 Power analysis**

Curves showing the results of simulations estimating the power to detect associations between antidepressant efficacy and metabolizer status in our study.
